# Chemical Modification of Polaronic States in Anatase TiO_2_(101)

**DOI:** 10.1021/acs.jpcc.1c03684

**Published:** 2021-06-24

**Authors:** Alex J. Tanner, Robin Kerr, Helen H. Fielding, Geoff Thornton

**Affiliations:** † Department of Chemistry, University College London, 20 Gordon Street, London WC1H 0AJ, United Kingdom; ‡ London Centre for Nanotechnology, University College London, 17-19 Gordon Street, London WC1H 0AH, United Kingdom

## Abstract

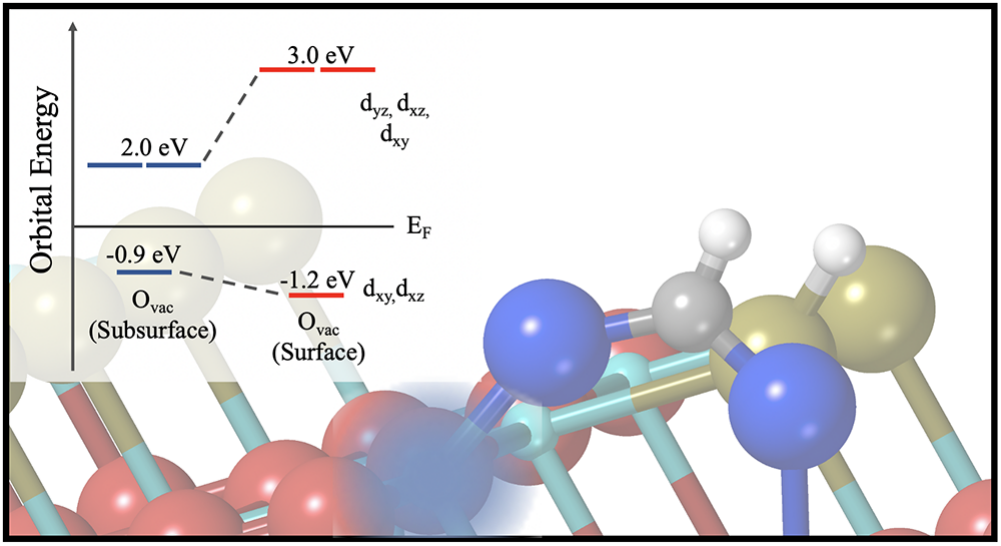

Two polymorphs of TiO_2_, anatase and rutile, are employed in photocatalytic applications. It is broadly accepted that anatase is the more catalytically active and subsequently finds wider commercial use. In this work, we focus on the Ti^3+^ polaronic states of anatase TiO_2_(101), which lie at ∼1.0 eV binding energy and are known to increase catalytic performance. Using UV-photoemission and two-photon photoemission spectroscopies, we demonstrate the capability to tune the excited state resonance of polarons by controlling the chemical environment. Anatase TiO_2_(101) contains subsurface polarons which undergo sub-band-gap photoexcitation to states ∼2.0 eV above the Fermi level. Formic acid adsorption dramatically influences the polaronic states, increasing the binding energy by ∼0.3 eV. Moreover, the photoexcitation oscillator strength changes significantly, resonating with states ∼3.0 eV above the Fermi level. We show that this behavior is likely due to the surface migration of subsurface oxygen vacancies.

## 1 Introduction

The polaronic Ti^3+^ states of TiO_2_ have long been a source of technological interest. They arise from the reduction of TiO_2_ and result in n-type semiconductor properties in the material. Not only do these excess electrons give rise to the conductivity that permits many surface studies, they also facilitate a wide range of redox chemistries such as water splitting and hydrogen generation.^1−3^ In recent years, it has been shown that these polaronic states, commonly referred to as the band gap states (BGS) of TiO_2_, can also undergo photoexcitation processes,^4−6^ potentially contributing to the catalytic photoyield. Of the two predominant TiO_2_ polymorphs, anatase demonstrates higher catalytic performance.^7^ Despite this, the polaronic states of anatase remain poorly understood. In part, this is because, unlike in rutile TiO_2_, anatase polarons rarely exist at the surface in ultrahigh vacuum (UHV) conditions, resulting in fewer microscopy studies and weaker spectroscopic signals.^8,9^

**Figure 1:**
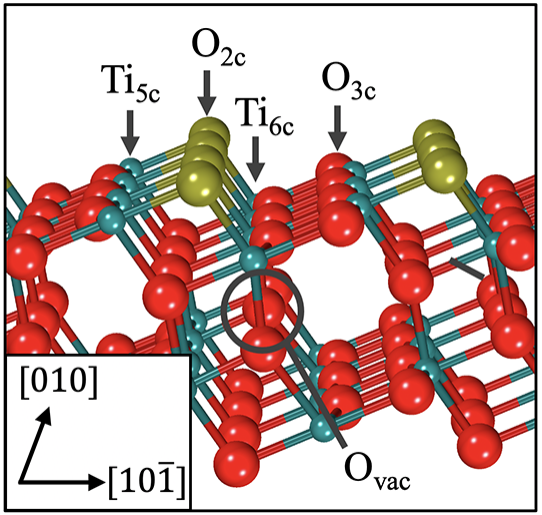
Ball-and-stick model of anatase TiO_2_(101). Turquoise spheres denote Ti ions. Red spheres represent O ions, with two-fold coordinated bridging O shaded gold. A subsurface oxygen vacancy (O_vac_) is labeled, which represents a common defect and localization point of polarons.

The (101) facet has the lowest surface free energy of anatase TiO_2_,^10,11^ adopting a (1 × 1) sawtooth structure (see Figure 1). This surface is composed of five- and six-fold coordinated Ti atoms (Ti_5c_ and Ti_6c_), bonded to three- and two-fold coordinated O atoms (O_3c_ and O_2c_), respectively. Preparation of this surface in UHV reduces the sample and gives rise to oxygen vacancies (O_vac_), which are well-known to reside in the subsurface (generally defined as 1 or 2 atomic layers below the surface).^9,12,13^ It has been proposed that this is due to greater atomic relaxations within this region, which results in a lower vacancy formation energy.^8,14^ Recently, the influence of the crystal field has also been suggested as a contribution toward subsurface O_vac_ formation, with a bulk-like octahedral field favorable for the resulting polarons.^15^

Spectroscopically, the BGS of TiO_2_(101) have been investigated using photoemission techniques, where they are detectable at ∼1.0 eV binding energy (BE).^16−18^ Formally, the BGS are Ti 3d in character, resulting from the Jahn–Teller splitting of d-orbitals in the *pseudo*-octahedral crystal field of anatase, which gives rise to orbitals of *t*_2*g*_- and *e*_*g*_-like symmetry.^16−18^ Two-photon photoemission spectroscopy (2PPE) is a pump-probe technique that offers additional information about the BGS, such as photoexcitation properties. 2PPE spectra are most commonly produced as a result of coherent (simultaneous two-photon excitation of an occupied state) or incoherent (two sequential one-photon excitations via an intermediate state) processes, providing information on both the occupied and the excited states, respectively. Although there have now been numerous 2PPE studies of rutile TiO_2_,^4−6,19−21^ few have been published on anatase.^22,23^ In these earlier studies, a relatively weak 2PPE resonance was observed for anatase TiO_2_(101), which was ascribed to excitation of the BGS to an excited state ∼2.5 eV above the Fermi level (*E*_F_). This is similar to the behavior observed for rutile TiO_2_(110).^22^

Polarons form at O_vac_ in both anatase and rutile TiO_2_, although their behavior differs distinctly in the two polymorphs. In rutile TiO_2_(110), polarons are known to have a low energy barrier for “hopping” to adjacent Ti atoms.^24−27^ Consequently, although the rutile UPS BGS intensity can fluctuate significantly with the surface environment,^28−30^ the BE of the initial and excited states only shifts minimally.^4,31^ In anatase, however, density functional theory (DFT) and scanning tunneling spectroscopy (STS) have shown that polarons associated with O_vac_ have higher energy barriers for hopping and hence are localized at the defect.^8,32^ This suggests that polarons in anatase are able to be trapped at specific defect sites when the surface is chemically altered, with the potential to modify the photoexcitation behavior. In this work, we test this hypothesis by forming two distinctive anatase TiO_2_(101) surface environments. As-prepared TiO_2_(101) with subsurface defects is denoted as C-A101, and TiO_2_(101) with a saturated coverage of dissociatively adsorbed formic acid is denoted FA-A101.

## 2 Methods

UV photoemission spectroscopy (UPS, VG Microtech) and 2PPE experiments were performed in a UHV system with a base pressure of ∼1.0 × 10^–10^ mbar, with the partial pressure of residual water ∼3 × 10^–11^ mbar. UPS and 2PPE spectra were recorded with a hemispherical electron energy analyzer (VG Scienta R3000) with the entrance lens normal to the sample surface, with the sample biased by −6.0 V. Photoemission from the Ta sample holder was used to determine the position of *E*_F_. UPS and 2PPE were both used to measure workfunction values. The incident angle of the laser was 68 ± 1° from the surface normal, with the laser spot having a diameter of ∼0.5 mm at the sample. The system is also equipped with an X-ray (VG Microtech) source, which enables core-level photoelectron spectroscopy (XPS) measurements. All spectra were recorded at room temperature (RT) unless otherwise indicated.

Tunable femtosecond laser pulses (280–390 nm) were generated by Light Conversion tunable optical parametric amplifiers (TOPAS-c), pumped by a Coherent Legend regenerative amplifier operating at 1 kHz, seeded by a Ti-sapphire oscillator (Coherent Micra). The power was reduced to ∼1 mW using neutral density filters to minimize space-charge effects. UPS measurements were performed to check for laser-induced defect states, with none being found. To ensure that the results were not influenced by fluctuations in laser power, it was monitored throughout experiments from a separate beam via a beam splitter earlier in the optical sequence after the TOPAS-c. The polarization of light relative to the crystal orientation is controlled by the use of a periscope that is inserted at the end of the optical sequence.

The natural anatase TiO_2_(101) crystal (MaTeck) and rutile TiO_2_(110) sample were cleaned with multiple cycles of 20 min sputtering (1 kV, 1 μA cm^–2^) and 10 min annealing at 950 and 1000 K, respectively. The sample temperature was monitored via a K-type thermocouple in close proximity to the sample, as well using a pyrometer (Minolta). Photoemission spectra of anatase TiO_2_(101) were taken after approximately 1 h to ensure full migration of surface O_vac_ to the subsurface. After cleaning, XPS evidenced a contamination level of ∼0.05 ML measured in the anatase sample, comprising naturally occurring Nb, Ca, C, and Na. XPS of the rutile sample evidenced a contamination of <0.05%. Both samples displayed a sharp (1 × 1) low energy electron diffraction (LEED) pattern. LEED was used to determine that the [010] azimuth lay in the horizontal direction of the anatase sample. Formic acid exposure was via gas phase dosing under UHV conditions from a liquid sample. Samples were cleaned via freeze/thaw pumping to remove dissolved O_2_ and CO_2_, which could affect accurate monitoring of the defect states. The purity of the acid in the gas phase was monitored by a residual gas analyzer (Hiden Analytical, HAL 101). The resulting interfaces were characterized by XPS.

## 3 Results and Discussion

### 3.1 Sub-Band-Gap Polaron Photoexcitation in C-A101

**Figure 2:**
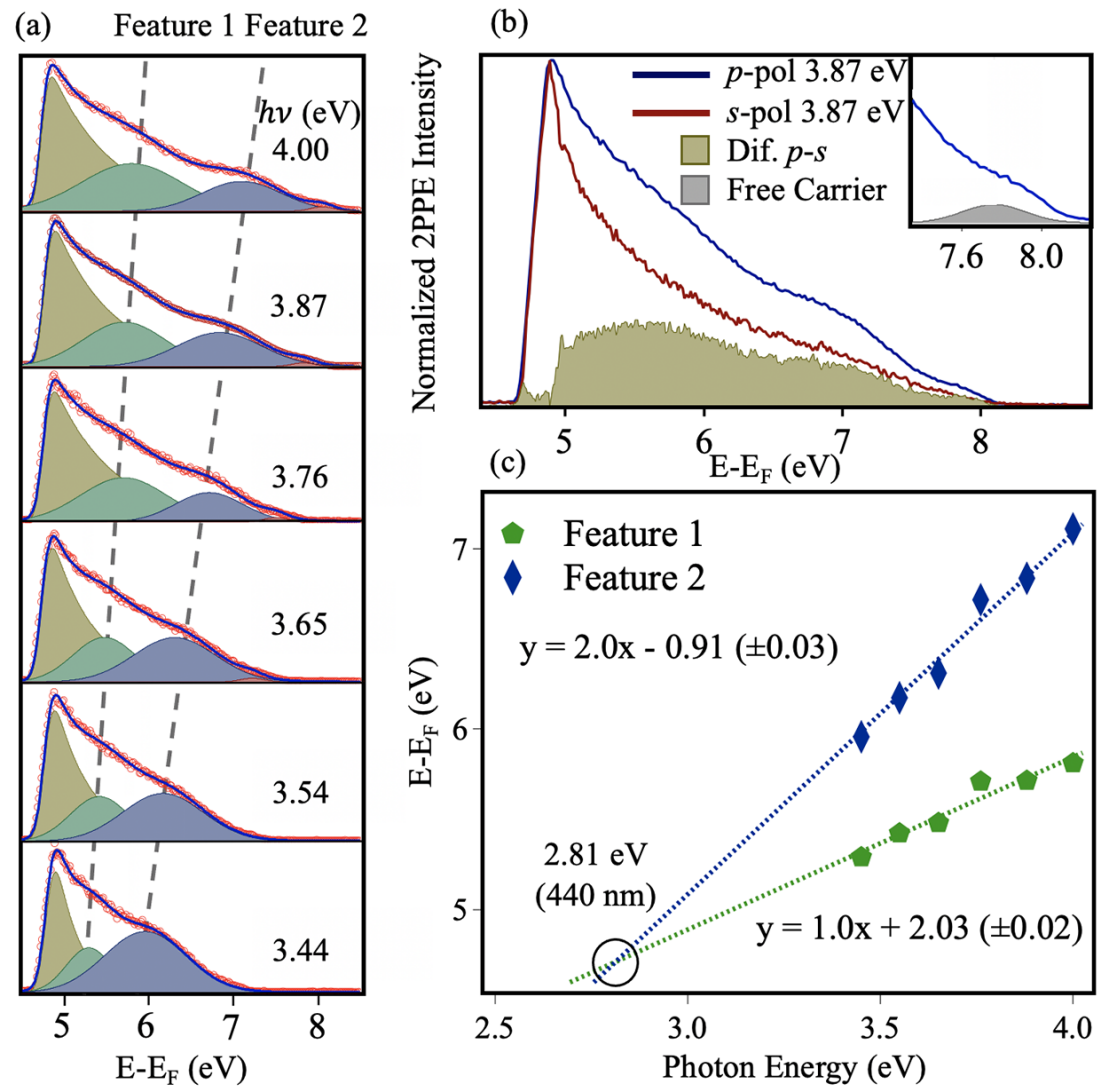
(a) 2PPE spectra (*h*ν = 3.44–4.00 eV, 360–310 nm) measured from C-A101 with *p*-polarized light. Spectra were fitted using the procedure described in previous work (see refs (20) and (21)). The red circles represent the original data points, and the blue line represents the fit. The gold peak represents the 2PPE background. Two dominant peaks are observed, labeled feature 1 and feature 2 (green and blue Gaussians, respectively). (b) 2PPE spectra (*h*ν = 3.87 eV, 320 nm) measured with *p*- and *s*-polarized light. The spectra are normalized to the intensity at the workfunction cut off. The difference spectrum (*p* – *s*) shows the presence of the two dominant features identified in (a), as well as a third feature at ∼7.7 eV from the free carrier population, which is enlarged in the inset. (c) Plot of the photon energy dependence of the two fitted peaks in (a). The gradient (*x*) and *y*-intercept values determine the 2PPE process and intermediate/initial state energy, respectively, according to equations for coherent (*E* – *E*_F_ = *h*ν_probe_ + *h*ν_pump_ + *E*_initial_) and incoherent (*E* – *E*_F_ = *h*ν_probe_ + *E*_intermediate_) excitations. A blue diamond signifies feature 2 (coherent) and a green pentagon, feature 1 (incoherent). The resonant photon energy (circled) is identified by the point at which the two lines intersect one another and is calculated as ∼2.81 eV (440 nm).

At the C-A101 surface, we investigated photoexcitation of polarons associated with subsurface vacancies.^33,34^ 2PPE spectra of as-prepared anatase TiO_2_(101) that contains subsurface vacancies are shown in Figure 2a. The red circles represent the raw data points, and the blue line represents the subsequent fit to two Gaussians and a background. Two features become apparent at higher *h*ν, labeled feature 1 and feature 2, which have a different electron energy dependence when varying the photon energy. The polarization dependence of the 2PPE spectra at 3.87 eV, 320 nm (Figure 2b) shows that the oscillator strength is higher with *p*-polarized light for all features, in agreement with previous work.^22^ Features 1 and 2 are visible as well as a third, smaller feature at 7.7 eV (see inset). Its energy suggests that it is a coherent 2PPE feature from the shallow donor state slightly below *E*_F_.^32,35^Figure 2c gives the quantitative representation of the final state energy (*E* – *E*_F_) dependence on photon energy (eV). In these plots, data points produce gradients of 1 or 2 for incoherent and coherent processes, respectively, with a *y*-intercept equal to the intermediate or initial state energy, respectively (see Figure 2 caption for equations). We find a coherent and an incoherent contribution, where the incoherent 2PPE process (feature 1) represents excitation into an intermediate state of *t*_2*g*_-like character,^22^ ∼2.0 eV above *E*_F_. Coherent 2PPE gives rise to feature 2, with a *y*-intercept of around −0.9 eV, consistent with excitation of BGS polarons. The resonant photon energy of the process is ∼2.81 eV, significantly less than that of the optical band gap, 3.20 eV.^7,33,34,36,37^ This is in line with previous studies that demonstrate the extended photoresponse of Ti^3+^ doped TiO_2_.^38,39^

**Figure 3:**
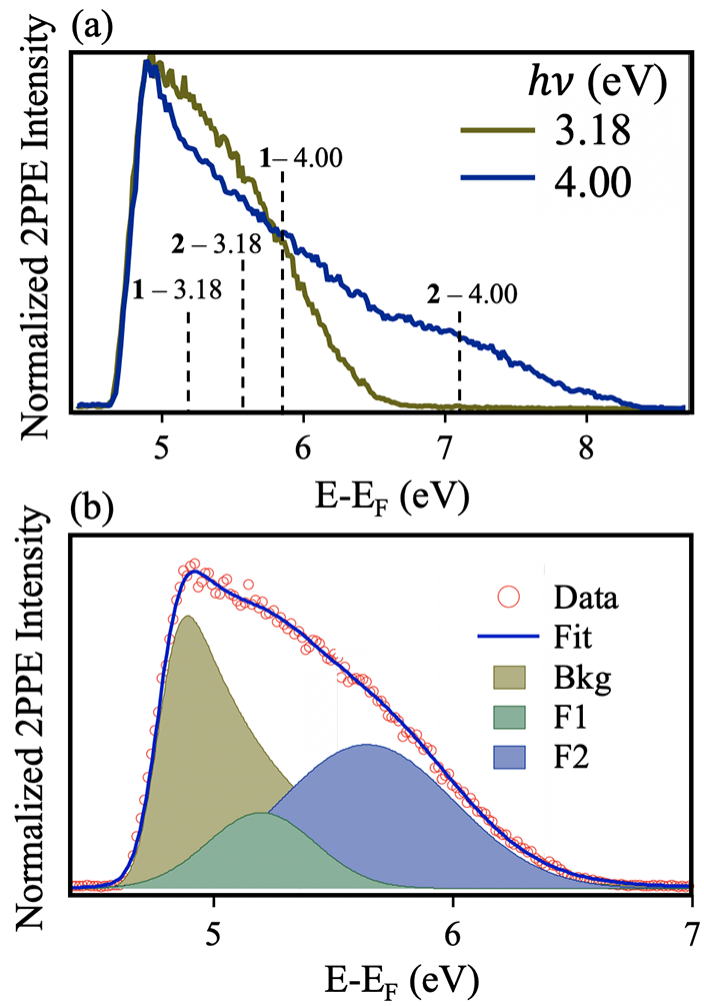
(a) 2PPE spectra of *h*ν = 3.18 eV (390 nm) and 4.00 eV (310 nm), with the peak locations for features 1 and 2 at each photon energy labeled (recorded with *p*-polarized light). The spectra are normalized at 4.89 eV (*E* – *E*_F_). (b) 2PPE spectrum (*h*ν = 3.18 eV, 390 nm) of C-A101 fitted with Gaussian peaks. The red circles represent the original data points, and the blue line is the fit to the background (Bkg), feature 1 (F1), and feature 2 (F2) contributions.

This interpretation differs from the assignment of spectra in earlier work,^22^ where it was concluded that the 2PPE spectra of C-A101 predominantly consists of an incoherent 2PPE feature via an intermediate state centered at 2.5 eV above *E*_F_, with no contributing coherent peak. The differing interpretation is likely a result of the available experimental photon energies. It is now clear that the photon energies employed in the previous work (*h*ν = ∼2.9–3.1 eV (430–400 nm)) are close to the resonant photon energy for BGS excitation in C-A101, resulting in significant overlap between the coherent and incoherent 2PPE feature. In our work, tunable optical parametric amplifiers (see Methods section) give access to higher photon energies, allowing the separation of features 1 and 2 in spectra. Figure 3a shows an example of this, which illustrates the overlap of normalized 2PPE spectra recorded at photon energies of 3.18 and 4.00 eV (390 and 310 nm). The locations of features 1 and 2 in the two spectra are labeled. The increase in spectral intensity at 5.3 eV in the 3.18 eV (390 nm) spectrum is due to greater overlap of the coherent and incoherent features at near resonant conditions. Figure 3b further demonstrates this by spectral fitting.

### 3.2 Defect Migration at the Formate TiO_2_(101) Interface

**Figure 4:**
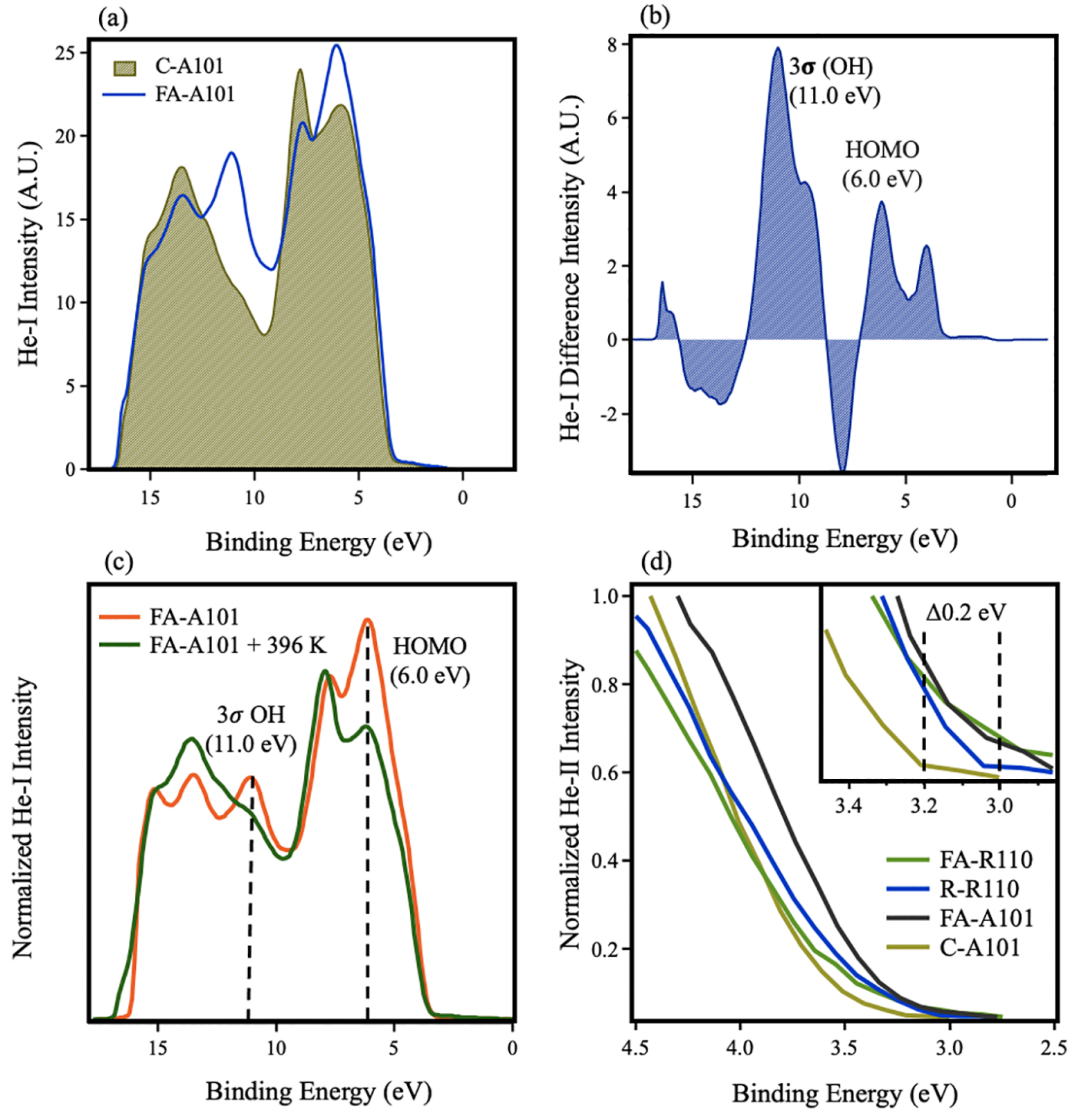
(a) He-I (21.2 eV) UPS spectra of C-A101 and FA-A101 following in situ gas phase dosing of formic acid. (b) Difference spectra from (a) showing peaks associated with the 3σ OH orbital and formate HOMO. Negative peaks represent a decrease in signal due to attenuation effects. (c) He-I (21.2 eV) UPS spectra of FA-A101 following flashing to 396 K and subsequent cooling to RT. Dashed lines represent the MOs of interest. (d) He-II (40.8 eV) spectra showing a comparison of the VBM of C-A101, FA-A101, R-R110, and FA-R110. The inset shows an expanded region near the VBM. Dashed lines represent the subsequent shift of the VBM. The secondary electron contribution has been removed with a Tougaard profile.

The adsorption of formic acid (HCOOH) on the anatase TiO_2_(101) surface has been studied experimentally and computationally in recent years.^40−44^ It is now known that formic acid adsorbs dissociatively on the anatase TiO_2_(110) surface at RT and saturates at ∼0.5 ML, as at the rutile TiO_2_(110) surface.^45^ However, in contrast to rutile (110),^46,47^ formate forms mixed monodentate/bidentate adsorption configurations at saturated adsorption coverages. Although these structural aspects are now clear, the effect of adsorption on the electronic structure is not known. Figure 4a shows unnormalized He-I (21.2 eV) UPS spectra of C-A101 and FA-A101, the latter being formed by in situ exposure to formic acid. Two additional features are evident at 6.0 and 11.0 eV BE following adsorption, which are also clear in the resulting difference spectrum (see Figure 4b). The peak at 11.0 eV is similar to a feature observed in spectra of rutile TiO_2_(110) surfaces after dissociative adsorption of water or carboxylic acids.^29,48−52^ This is assigned specifically to the 3σ OH orbital based on UPS studies of hydroxylated and trimethyl acetic acid TiO_2_ systems.^53,54^ The residual peak at 11.0 eV BE in the C-A101 spectrum has previously been attributed to small levels of water dissociation at step edges.^23^ In the difference spectrum, the peak at ∼4 eV arises predominantly due to an upward shift in the valence band maximum. This is associated with band bending caused by increased negative charge at the surface, which is expected to arise from O_vac_ and polaron migration to the surface (see below). The peak at ∼6.0 eV is best assigned to the formate highest occupied molecular orbital (HOMO) due to its similar appearance in the UPS spectra of formate on rutile (110).^55,56^

Following flashing FA-A101 to 396 K, we find that both the 6.0 and the 11.0 eV peaks are removed, and the spectrum largely resembles that of C-A101 (see Figure 4c). This clear desorption of formate is in agreement with recent thermal programmed desorption (TPD) and vibrational spectroscopy measurements, which evidenced the evolution of gas phase water at ∼400 K.^45^

Figure 4d compares the valence band maxima (VBM) of C-A101 and FA-A101 and the analogous rutile TiO_2_(110) equivalents with He-II (40.8 eV) UPS. Here, R-R110 refers to the as-prepared reduced surface which contains surface oxygen vacancies. FA-R110 denotes the formate saturated surface. The spectra are shown after removal of the secondary electron background with a Tougaard function.^57^ As expected, the VBM of C-A101 and R-R110 occurs at ∼3.2 and 3.05 eV BE, respectively.^36^ Following a saturation adsorption of FA, the VBM of rutile TiO_2_(110) does not change. However, in anatase TiO_2_(101), the VBM shifts 0.2 eV lower in BE, a result of band bending caused by the migration of localized O_vac_ polarons to the surface.

**Figure 5:**
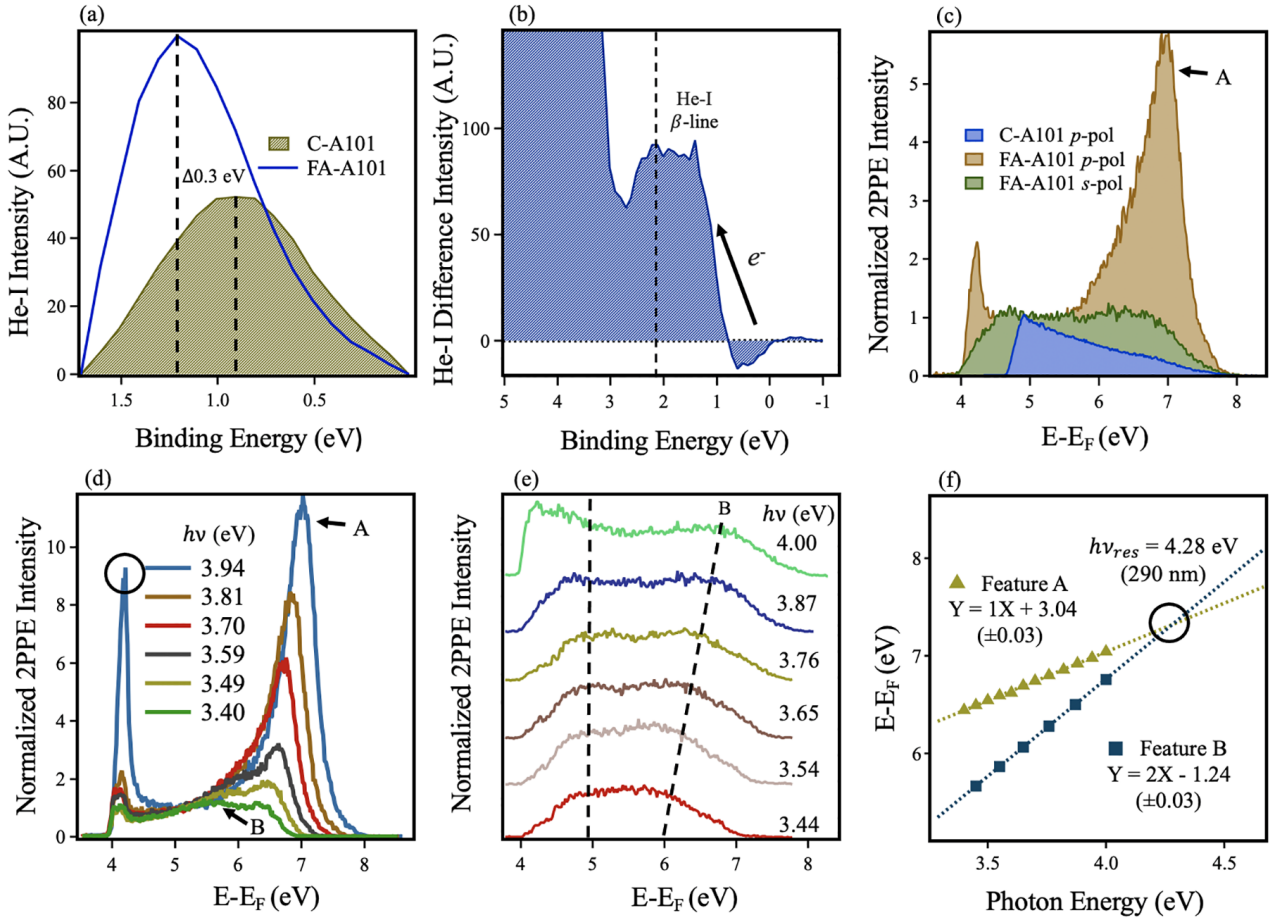
(a) BGS region of C-A101 and FA-A101 following exposure to formic acid via in situ gas phase dosing. Peaks are isolated following subtraction of the secondary electron background via a Tougaard function. Dashed lines represent locations of peak maxima. Following adsorption, the peak is 0.3 eV higher in BE and 63% larger by peak area. (b) Difference spectrum between the raw UPS He-I spectrum of FA-A101 and C-A101. The arrow represents the shift in electron density. The dashed line represents the maximum point of He-I β contribution from the valence band region. (c) 2PPE spectra of C-A101 and FA-A101 (*h*ν = 3.87 eV (320 nm)) normalized at 5.2 eV (*E* – *E*_F_). The polarization of light is shown in the panel legend. (d) 2PPE spectra of FA-A101 (*p*-polarized, *h*ν = 3.94–3.40 eV (315–365 nm)) showing the formation of a resonance peak in the 2PPE spectra. Spectra have been normalized at 5.2 eV (*E* – *E*_F_). The circle represents the increased 2PPE signal from coherent 2PPE valence band contributions at higher *h*ν. (e) Stacked 2PPE spectra (*s*-polarized, *h*ν = 4.00–3.44 eV (310–360 nm)). Dashed lines represent two features: one lies at a constant *E* – *E*_F_ regardless of *h*ν, the other the movement of feature B with *h*ν. (f) The gradient (*x*) and *y*-intercept values determine the 2PPE process and intermediate/initial state energy, respectively, according to equations for coherent (*E* – *E*_F_ = *h*ν_probe_ + *h*ν_pump_ + *E*_initial_) and incoherent (*E* – *E*_F_ = *h*ν_probe_ + *E*_intermediate_) excitations. A blue square signifies feature B (coherent) and a yellow triangle, feature A (incoherent). The circle represents the resonant photon energy for the excitation process.

Formation of FA-A101 causes the BGS peak to shift to ∼0.30 eV higher BE and to increase in intensity by 63% compared with C-A101 (see Figure 5a). UPS is a surface sensitive technique, probing the top ∼1 nm of the sample;^58^ this rise in BGS intensity is therefore evidence of an increased density of polarons in this region. This is in line with results from FA-R110,^20^ a key difference being that a BGS BE shift is not observed for FA-R110, whereas it is for FA-A101. This indicates that the polaronic states are in an altered chemical environment in FA-A101 compared with C-A101. The difference spectrum (see Figure 5b) shows a decrease in intensity between ∼0 and 0.9 eV BE following formic acid adsorption, evidencing a movement of the original C-A101 polaron population. An explanation for these observations is that subsurface O_vac_ (and associated polarons) are diffusing to the surface, as has been suggested by both IRRAS and DFT calculations.^41,45^ The shift of 0.3 eV BE is also in agreement with measurements by Setvin et al., where the BE's of subsurface and surface polarons were measured by UPS and STS, respectively, following tip-induced migration of O_vac_ (and polarons).^32^ Furthermore, DFT calculations of FA-R110 showed no density of states (DOS) in this region, excluding the possibility of formate molecular orbitals contributing to this signal.^20^ This adsorbate-induced migration likely occurs due to surface relaxations, which may reduce the energetic gain required to accommodate O_vac_ at the TiO_2_(101) surface.^8,14^ This may be related to the significant relaxation at TiO_2_ surfaces caused by formic acid adsorption.^45,59^

**Figure 6:**
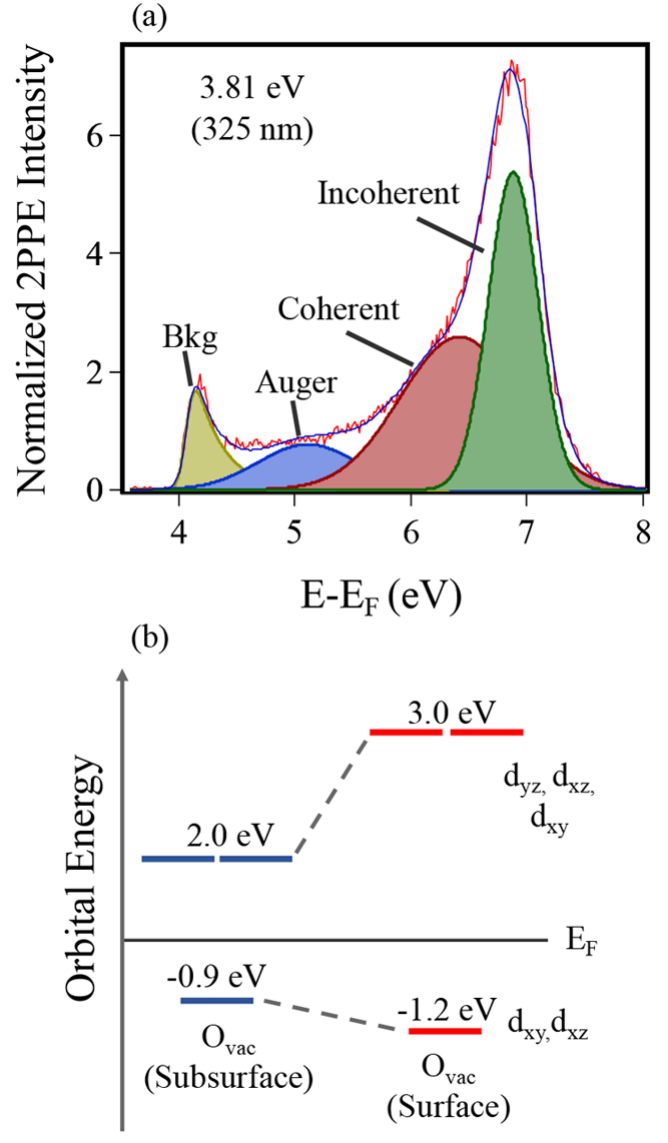
(a) 2PPE *p*-polarized spectrum (*h*ν = 3.81 eV, 325 nm) of FA-A101 fitted with Gaussian lineshapes. The red line represents the original spectrum, and the blue line represents the fit to the Gaussian distributions. The gold peak represents the 2PPE background. Three dominant features are labeled corresponding to their 2PPE origin. (b) Orbital energy level diagram demonstrating the orbital splitting and resonance levels of polarons in C-A101 (subsurface O_vac_) and FA-A101 (surface O_vac_). Excited state energies are obtained from 2PPE. The occupied state energies are obtained from UPS.

2PPE spectra of TiO_2_(101) also show substantial differences following formic acid adsorption (see Figure 5c). In the *p*-polarized spectrum specifically, FA-A101 displays a large peak at ∼6.9 eV (*E* – *E*_F_). On varying the photon energy, this peak (labeled feature A) shifts in energy and displays a strong intensity dependence (see Figure 5d). A smaller, broader feature, B, also becomes clear at lower photon energies and appears to be merging with feature A, indicative of a resonance process.^60^ By comparing *p*- and *s*-polarized spectra, it is evident that feature B is present in both. Figure 5e shows the *s*-polarized 2PPE spectra as a function of *h*ν. Feature B clearly shifts in energy but displays little intensity dependence. Another feature at lower energy shows no *h*ν dependence, which is likely the result of an Auger process, as has been observed in the 2PPE spectra of FA-R110.^20^Figure 5f shows the *E* – *E*_F_ versus *h*ν dependence of features A and B taken from *p*- and *s*-polarized 2PPE spectra, respectively. We find that feature A is produced by an incoherent 2PPE process from an intermediate state centered 3.04 eV above *E*_F_, whereas feature B is a coherent process from the FA-A101 BGS at ∼1.2 eV BE. The 2PPE features from FA-A101 are labeled in Figure 6a, which shows the *p*-polarized spectra (3.81 eV, 325 nm) following peak fitting. In previous work, DFT was used to calculate the density of states (DOS) of Ti^3+^ electrons at a variety of O_vac_ positions in TiO_2_(101); the intermediate state energy reported here matches the position of surface-localized DOS in the conduction band.^15^ These DOS are *t*_2*g*_-like in character, suggesting a *t*_2*g*_ → *t*_2*g*_ transition, as in C-A101. The orbital energy diagrams for BGS polarons and their resonant excited state for C- and FA-A101 are shown in Figure 6b. The formation of a large 2PPE feature supports the interpretation from UPS that surface migration of polaronic states occurs upon formic acid adsorption; the large shift in resonance photon energy also demonstrates the changing chemical environment. As a result of the new coordination environment, the *t*_2*g*_-like orbitals of surface-localized O_vac_ polarons have an increased energy separation. Polarons in TiO_2_ exist in distinct configurations, dictated by the electric field of the surface.^61^ The clear dependence of the photoexcitation on the electric field vector orientation reveals that O_vac_ polarons at the FA-A101 surface largely exist in a single configuration, with a transition dipole moment in the [010] direction.

Although we have focused on formate adsorption in this work, the adsorbate-induced modification of polaronic states in TiO_2_(101) is likely observable in other systems with high surface coverages and significant surface relaxations. It may be possible to exert further control over this effect based on the electrostatic properties of the adsorbate.

## 4 Conclusions

In summary, we have shown that it is possible to tune the energetics of O_vac_ polarons in anatase TiO_2_(101) by chemically altering the surface environment. It is likely that this control is possible due to the decreased mobility of polarons at anatase O_vac_ sites, relative to those in rutile. On C-A101, subsurface polarons undergo resonant photoexcitation at energies below that of the band gap, potentially giving rise to an extended photoresponse. UPS confirms that formic acid adsorbs dissociatively on TiO_2_(101). The effect on the BGS is profound, shifting the BE 0.3 eV higher and causing the formation of a large, sharp resonance peak in the 2PPE spectra. This peak is a result of strong coupling from the BGS to an intermediate state 3.04 eV above *E*_F_, which also evidences an increased splitting of the *t*_2*g*_-like energy levels. Our UPS and 2PPE studies indicate that this modification is caused by surface O_vac_ and polaron migration, as was also suggested in vibrational spectroscopy measurements.^41^

Manipulating polaronic states has mostly been limited to STM tip engineering. Here, we demonstrate the ability to do so on the macroscale through a facile chemical process. This capacity to tune the energetic environment of polarons has implications for the technological applications of anatase TiO_2_ that look to exploit the role of polaronic states.
